# Can Machine Learning Help Us Understand Social Connection and Its Impact on Health?

**DOI:** 10.1001/jamanetworkopen.2024.51545

**Published:** 2024-12-02

**Authors:** Thomas K. M. Cudjoe, Ashwin A. Kotwal

**Affiliations:** Division of Geriatric Medicine and Gerontology, Department of Medicine, Johns Hopkins University School of Medicine, Baltimore, Maryland; Division of Geriatrics, Department of Medicine, University of California, San Francisco; Geriatrics, Palliative, and Extended Care Service Line, San Francisco Veterans Affairs Medical Center, San Francisco, California

The role of social connection in influencing health outcomes has become an increasingly discussed topic globally for people across the lifespan. A prescient report by the National Academies of Sciences, Engineering, and Medicine (NASEM) released in February 2020 outlined the need for health systems to address loneliness and social isolation among older adults.^1^ And very soon after that, a global pandemic ensued, making experiences of social isolation and loneliness commonplace.^2^ As we emerge from the pandemic, attention on this subject continues, with the 2023 Surgeon General Advisory and the World Health Organization Commission on Social Connection calling for cross-sector collaboration and interventions to enhance social connection. Personalized strategies that account for the nuanced differences in people’s social lives and address their particular needs are necessary.

Elsewhere in *JAMA Network Open*, Bu and Fancourt^3^ advance our understanding of how social connection can be further characterized using machine learning and how these clusters impact health outcomes. They used data from a nationally representative sample of 7706 individuals (aged ≥50 years) in England from 2008 to 2009 to 2010 to 2011.^3^ The survey contained detailed questions on different dimensions of social connection, including (1) structure (size and diversity of the network), (2) function (how well social relationships meet emotional and practical needs), and (3) quality (positive and negative perceptions of relationships). These 3 dimensions informed by the Holt-Lundstad et al^4^ framework were highlighted in the 2020 NASEM report, are an instrumental step toward a comprehensive understanding of social connection. Their analysis revealed the presence of 5 clusters, ranging from highly connected (33% of the sample), where each of the 3 dimensions was strong, to disconnected (13% of the sample), where each dimension was poor. The remaining clusters were in between these 2 extremes; 2 clusters had gapped structure (referring to limited but present social ties) with either high function (21% of the sample) or low function (14% of the sample), and 1 cluster had a strong structure but poor function and mixed quality (20% of the sample).

Perhaps unsurprisingly, their analysis reveals that people in the highly connected cluster tended to experience better outcomes compared with those in the disconnected clusters. Less expected was that all clusters between these extremes had worse health than the highly connected cluster across mental well-being, physical health, and life satisfaction. The authors draw particular attention to the cluster that maintained structural aspects of social connection but had poor function and mixed quality, which had markedly worse health for each of the 5 health outcomes. In other words, simply having an extensive network is insufficient for promoting good health if the relationships within that network lack emotional fulfillment or involve negative interactions (poor quality). Amid all of this, causal inference studies are needed to expand our understanding of these relationships.

A central takeaway from the study by Bu and Fancourt^3^ is the role of integration of both subjective and objective indicators of social connection across the 3 different dimensions. While some prior studies solely focus on social isolation or loneliness, essential to distinguish, this work takes this further. The authors’ multidimensional view of social connection acknowledges that the quantity and quality of social ties and their functional aspects contribute to health outcomes. This work elucidates certain profiles or clusters that may be appropriate for specific interventions but not others. For example, interventions aimed at improving the function and quality of existing relationships might be more suitable for individuals in the gapped structure/poor function cluster (eg, psychotherapy). In contrast, those in the disconnected group may benefit more from strategies that address all 3 dimensions (eg, group-based or peer interventions that nurture bridges between people and support them as they navigate maladaptive social cognition). Future research should investigate whether these clusters remain stable over time or if individuals shift between clusters with changing circumstances. Such work could expand insights about the dynamics and intensity of social connection and how these patterns evolve, potentially guiding more personalized intervention strategies.^5^ This work may also help refine intervention efforts, as certain clusters might respond differently to specific interventions. Additionally, comprehensive social measures in several national surveys allow this machine learning approach and examination of these clusters to be replicated.^6^

A second consideration is the relevance of these clusters to clinical care, health systems, or community organizations. Clinicians are increasingly tasked with more screenings.^7^ Thus, this study’s nearly 50 questions on social life may be impractical in certain settings. How can these differences in social life be captured? More research is needed on the best setting (health systems, community, or both) to administer such surveys. How do we efficiently capture the correct information and ensure that social connection interventions are evaluated with the optimal outcome measure? Clinical and community practitioners need tools that help them efficiently and effectively identify the presence of social isolation or loneliness so that they can act. Future research might determine the correct balance between detailed measures of social connection vs brief instruments to identify these clusters in clinical or other settings. This could facilitate early intervention and reduce the associated burden.

A third consideration is the generalizability of study findings to underrepresented groups. This is of concern because many individuals did not complete specific survey measures and are thus not included in the analysis. This is also true with many national survey studies; certain population factors that extend from race, ethnicity, and socioeconomic status to physical or cognitive function or some other relevant status may be excluded from analysis or not recruited for the study, and, in this case, these clusters. Social connection may also be impacted by where an individual lives and their personal history or proximity to substance use, mental illness, or cognitive impairment. Understanding how their experiences differ from those identified in this current study may expand our knowledge about each of these clusters.

In conclusion, the growing body of research on social connection underscores its critical importance for public health, particularly as we continue to emerge from a global pandemic that heightened our focus on social isolation and loneliness. Moving forward, the study by Bu and Fancourt^3^ demonstrates that it is essential that we take a multidimensional approach to social connection, both in terms of measurement and intervention design, to ensure that we address the diverse needs of populations at risk. By understanding the differences in how and why individuals connect with others, we can better target interventions to those most at risk, ultimately promoting healthier, more connected populations.
