# Error in Funding

**DOI:** 10.1001/jamanetworkopen.2025.2071

**Published:** 2025-02-21

In the Invited Commentary, “Can Machine Learning Help Us Understand Social Connection and Its Impact on Health?”^1^ published December 23, 2024, there was an error in the Funding/Support section. Dr Cudjoe’s support from the Johns Hopkins University Center for Innovative Medicine Human Aging Project as a Caryl & George Bernstein Scholar was omitted. This article has been corrected.^1^
